# Integrating a Potentiometer into a Knee Brace Shows High Potential for Continuous Knee Motion Monitoring

**DOI:** 10.3390/s21062150

**Published:** 2021-03-19

**Authors:** Christin Büttner, Thomas L. Milani, Freddy Sichting

**Affiliations:** Department of Human Locomotion, Chemnitz University of Technology, 09107 Chemnitz, Germany; thomas.milani@hsw.tu-chemnitz.de (T.L.M.); freddy.sichting@hsw.tu-chemnitz.de (F.S.)

**Keywords:** wearable sensor technology, continuous monitoring, daily activities, knee injury

## Abstract

Continuous monitoring of knee motion can provide deep insights into patients’ rehabilitation status after knee injury and help to better identify their individual therapeutic needs. Potentiometers have been identified as one possible sensor type for continuous monitoring of knee motion. However, to verify their use in monitoring real-life environments, further research is needed. We aimed to validate a potentiometer-embedded knee brace to measure sagittal knee kinematics during various daily activities, as well as to assess its potential to continuously monitor knee motion. To this end, the sagittal knee motion of 32 healthy subjects was recorded simultaneously by an instrumented knee brace and an optoelectronic reference system during activities of daily living to assess the agreement between these two measurement systems. To evaluate the potentiometer’s behavior during continuous monitoring, knee motion was continuously recorded in a subgroup (*n* = 9) who wore the knee brace over the course of a day. Our results show a strong agreement between the instrumented knee brace and reference system across all investigated activities as well as stable sensor behavior during continuous tracking. The presented potentiometer-based sensor system demonstrates strong potential as a device for measuring sagittal knee motion during daily activities as well as for continuous knee motion monitoring.

## 1. Introduction

Knee ligament injuries are the most common knee injuries [1]. In addition to their acute impact on health, knee ligament injuries are potential risk factors for later knee osteoarthritis [2] and chronic balance impairments [3]. Evidence suggests that early-stage recovery is important for restoration of joint stability and prevention of knee disabilities in late life [4,5,6]. Sagittal knee range of motion (ROM), in particular, is frequently impaired after knee ligament injury and can impact the execution of daily activities both in the short- and long-term [4]. Its continuous monitoring during the early phase of rehabilitation could allow physicians and clinicians to better identify individual therapeutic needs for patients.

Recently, patient monitoring has gained importance in rehabilitation and clinical research [7]. By offering additional and more extensive insights into patients’ rehabilitation statuses, patient monitoring has the potential to complement existing measures, such as questionnaires, ROM measurements, functional tests, or conventional gait analysis, for assessing knee joint functionality. Most of these existing methods can only capture a snapshot of the patients’ status, which may not reflect the patient’s natural movement behavior. Patient monitoring, on the other hand, could allow a continuous and more detailed reflection of the knee movement. It may enable the detection of rare events or individual characteristic movement patterns, which may not be observed during brief assessments [7]. Thus, continuous monitoring could represent a keystone to new scientific approaches in rehabilitation. A crucial first step towards this goal is developing continuous knee motion monitoring systems and evaluating their reliability for measurements in real-life environments.

Despite the possible advantages of knee motion monitoring in research and rehabilitation, identifying suitable sensors for that purpose remains challenging. Several wearable sensors have been identified for continuous monitoring of knee motion, including potentiometers, flexible electrogoniometers, accelerometers, inertial measurement units (IMUs), and textile-based sensors [8,9]. But those sensors face technological and physiological challenges that can influence their performance and accuracy during monitoring. From our perspective, great challenges for wearable sensors include sensor drift, unphysiological data outputs, and/or even sensor breakdown. The latter can be caused by temperature changes, moisture, clothing, individual anthropometry, and sensor attachment. Another challenge relates to the large variability of knee motion during daily life. Sagittal knee angles can range from −10° extension to 160° flexion [10] during various activities of daily living (ADLs), including ground walking, stair walking, getting up from a chair, or jogging. During those activities, the sagittal knee motion varies in speed and acceleration depending on the individual’s physical limitations.

Another critical point to consider is patient compliance, especially when people are meant to be equipped with sensor systems over the course of multiple hours or days. To this end, it is crucial that the sensor systems do not interfere with clothing or restrict performance of daily activities. In addition, they should be easy to use. Recent research shows that patient compliance for monitoring systems can be increased by embedding sensors into clothing or clothing accessories, as it eliminates the need to remember to attach the sensors [11,12].

Considering these technological and physiological challenges, a potentiometer might fulfill many of the aforementioned requirements and could therefore be a promising sensor choice for continuous knee motion monitoring. This type of sensor is available in small sizes of a few centimeters in diameter and can withstand several million movement cycles before showing signs of wear and tear. Potentiometers have already been used for biomechanical analysis of the knee in clinical and daily settings [13,14,15,16,17,18], including the investigation of daily activities such as walking, jogging or stair climbing. In these studies, the potentiometer was typically attached to the lateral side of the leg with its fixed rotation axis aligned with the knee rotation axis and often embedded into knee braces or knee sleeves.

Even though potentiometers have been used to investigate sagittal knee motion in previous studies, remarkably few reports have validated this sensor type and compared it to a gold standard reference system for measuring joint kinematics (e.g., optoelectronic systems). Therefore, further research is needed to validate potentiometers for the measurement of sagittal knee motion during various activities. In addition, the question of a potentiometer’s applicability for continuous monitoring in real-life environments remains open. In this context, the objective of this study is to validate a potentiometer as an integrated component of a knee brace to measure sagittal knee motion during various ADLs in short- and long-term use. We thereby aim to evaluate the potentiometer’s potential as an instrument for continuous monitoring of sagittal knee motion.

## 2. Materials and Methods

### 2.1. Participants

Thirty-two healthy subjects (age: 27.5 ± 7.5 years; weight: 69.9 ± 10.8 kg; height: 172.2 ± 9.6 cm; 17 female, 15 male) participated in the study. The study complies with the regulations of the Declaration of Helsinki and the local faculty ethics committee expressed no concerns with the study method (V-333-17-TM-Validierung-14052019). All participants provided their verbal and written consent.

### 2.2. Instruments

**Figure 1:**
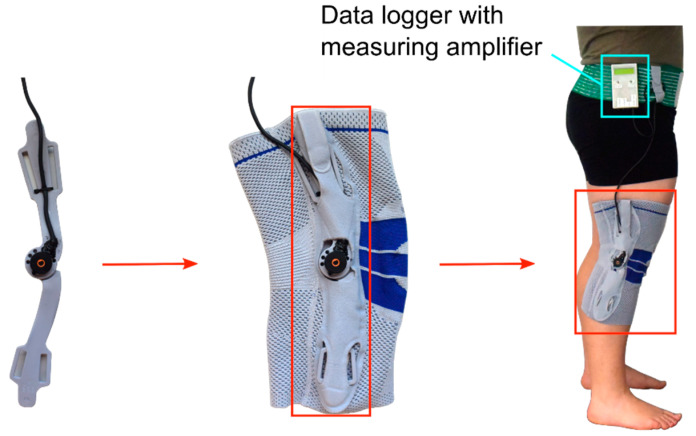
Implementation of the potentiometer to the lateral side of a conventional knee brace as indicated by the red arrows and boxes; the instrumented knee brace is connected to a data logger via cable as indicated by the blue box.

A potentiometer (MHP24, Megatron Elektronik, Putzbrunn, GER) with a diameter of 24 mm and a fixed rotation axis was embedded into a conventional knee brace (GenuTrain S, Bauerfeind AG, Zeulenroda-Triebes, GER) to measure sagittal knee motion. The instrumented knee brace was connected to a data logger via cable, which was attached at the hip (Figure 1). The chosen knee brace is frequently prescribed for patients with knee ligament injury to enhance joint stability, reduce pain and swelling, as well as for functional improvement. Seven different sizes of the knee brace were available for this study, fitting people with lower and upper leg circumferences of 28 cm to 49 cm and 38 cm to 59 cm, respectively. The length of the knee brace ranged between 31 cm and 35 cm depending on its size. The appropriate size of the knee brace was determined by measuring the leg circumferences for each participant according to the manufacturer’s instructions.

An optoelectronic system (Vicon Motion Systems Ltd., Yarnton, UK) with eight cameras served as the reference system. For this purpose, twenty markers were attached to each participant’s lower body according to the Vicon PlugInGait Lower Body Ai Functional model. The system and marker model were selected for their reliability and validity [19].

### 2.3. Sensor Calibration

**Figure 2:**
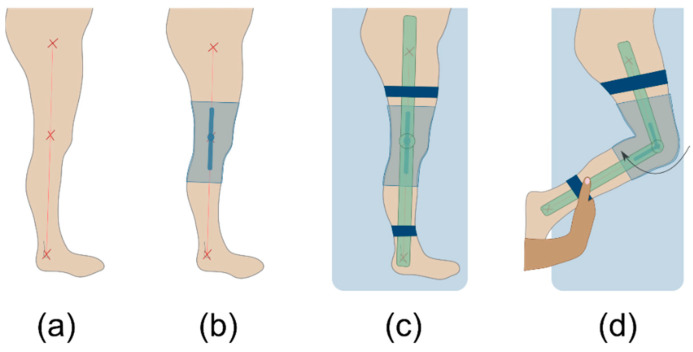
The four main steps of the calibration process; (**a**) marking and connecting anatomic landmarks, (**b**) putting on the instrumented knee brace, (**c**) attachment and alignment of the additional calibration goniometer in prone position, (**d**) passive knee movement in prone position by the investigator.

**Figure 3:**
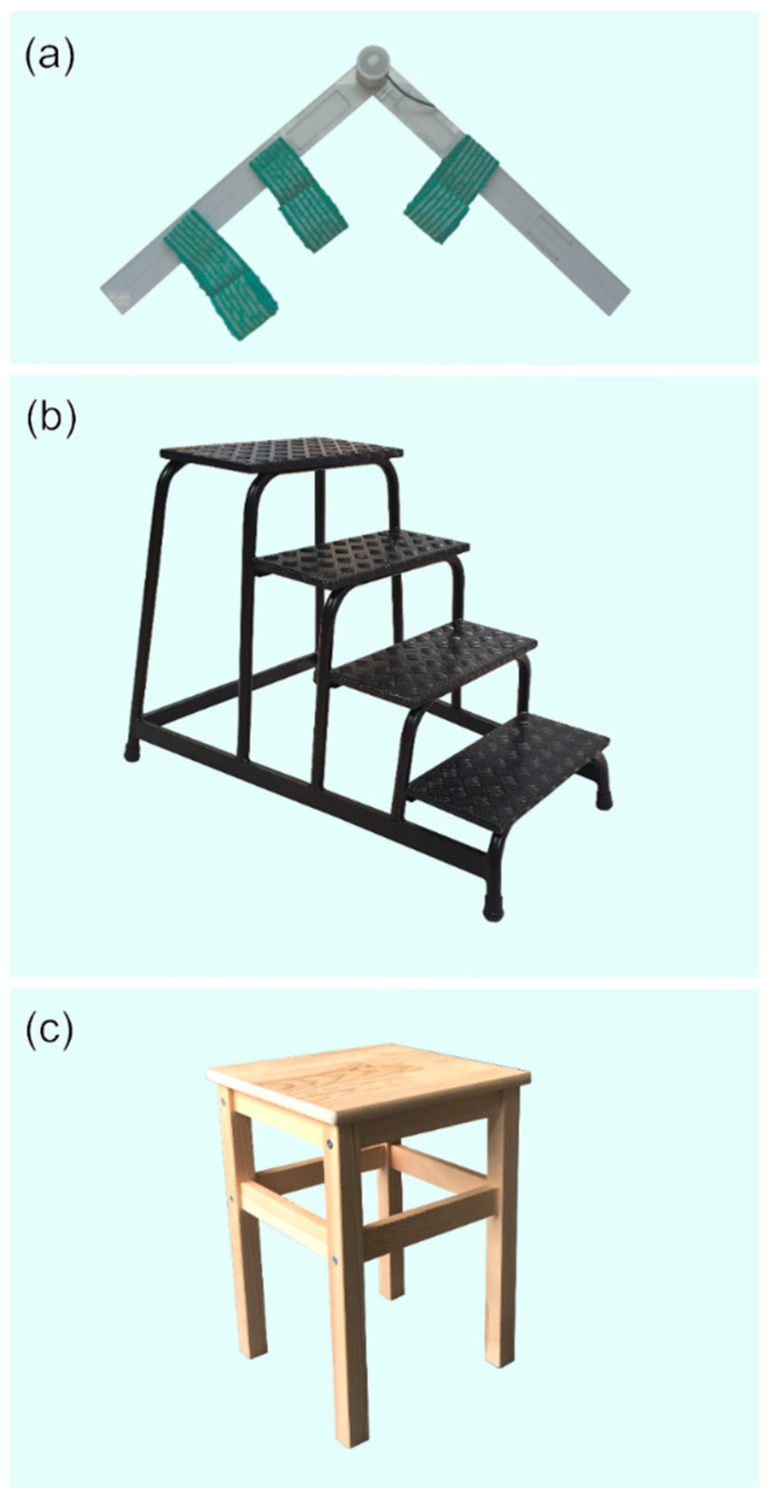
(**a**) Additional goniometer used during the calibration process, with Velcro straps and transparent lever arms that enabled positioning along anatomical lines, (**b**) portable aluminum staircase used for stair ascend and stair descend, (**c**) armless wooden stool used for sitting down and getting up.

The investigator calibrated the instrumented knee brace for each participant before the initial data collection. The calibration included four main steps (Figure 2). (a) First, the investigator palpated the trochanter major, knee rotation center, and lateral malleolus on the patient’s right leg and marked them with a skin marker (edding 8020, edding Vertrieb GmbH, Wunstorf, GER). After that, the anatomic lines between those anatomic landmarks were drawn on the patients’ leg. (b) Subjects then put on the instrumented knee brace with the sensor’s rotation axis aligned concentrically with the knee rotation axis. They were subsequently asked to lay on a massage table on their left side with their right leg straightened. (c) In this position, an additional electrical goniometer (EUP1425-1K, ETI Systems, Carlsbad, CA, USA) for measuring joint angles was attached to the lateral side of the extended leg. The lever arms of the additional goniometer were made of transparent plastic to guarantee the visibility of the lines between the anatomical landmarks (Figure 3a). The goniometer was attached to the patient’s leg with Velcro straps to reduce shift between the instrumented knee brace and the additional goniometer. The investigator then aligned the additional goniometer’s rotation axis and the lever arms with the rotation axis of the instrumented knee brace’s sensor and the anatomical lines, respectively. (d) In the final step, the investigator flexed the right knee until the participant’s maximum passive ROM was reached. During that motion, the knee angle data from the electrical goniometer and the raw data from the instrumented knee brace were recorded. The recorded data were exported and used to create an individual sensor fitting curve for each subject by interpolating a third-degree function. The individual functions were later used to convert the raw data of the instrumented knee brace into degrees.

### 2.4. Measurement Protocol

After calibration, participants were given some time to accustom themselves to the instrumented knee brace. Then, participants performed seven ADLs, including walking, jogging, stair ascent, stair descent, chair rising, sitting down, and squatting, in that order. One successful trial per activity was recorded with participants being barefoot and moving naturally at their own pace. After each trial, the recording was stopped. During both sessions, the corresponding sagittal knee motion was recorded synchronously by both the instrumented knee brace and the reference system. The instrumented knee brace and reference system recorded at a rate of 2000 Hz and 250 Hz, respectively. For further analysis, one complete movement cycle per activity and participant was extracted from the data of the right leg. All tests were conducted by one investigator.

### 2.5. Experimental Procedures

To assess the short-term agreement between the instrumented knee brace and reference system, all participants (*n* = 32) followed the measurement protocol once and thereby completed one measurement session (S1). To determine the long-term agreement between the instrumented knee brace and the reference system, a subgroup of nine participants completed a second session (S2) after continuously wearing the knee brace for a minimum of six hours. Session 2 followed the same protocol as S1, but the sensor calibration was only performed before S1. Knee angle data were recorded continuously between S1 and S2. The nine participants followed their daily routines, such as office work, yoga, gardening, and cycling. Contact sports and watersports (e.g., soccer and swimming) were not allowed due to safety reasons. The continuously recorded knee angle was used to verify the sensor behavior during continuous tracking. At the end of S2, the subgroup also evaluated their user experience with the instrumented knee brace. Participants filled out the Quebec User Evaluation of Satisfaction with assistive Technology (QUEST) questionnaire [20] and reported their perceived limitation in everyday life on a visual analog scale (VAS). The VAS ranged from zero to 100 with zero having no limitation at all and 100 being completely limited in everyday life due to wearing the instrumented knee brace.

### 2.6. Data Processing and Analysis

Data processing was performed using Vicon Nexus 2.8.1 (Vicon Motion Systems Ltd., Yarnton, UK) and Matlab R2018a (The MathWorks, Inc., Natick, MA, USA). In Nexus, sagittal knee angles from the reference system were processed using built-in pipelines, including a Woltring filter with a smoothing factor of 5. A customized Matlab script was used to process the data of the instrumented knee brace. These data were filtered using a 4th order Butterworth filter with a 50 Hz cut-off frequency. As datasets with equal length were needed for further data analysis, data of the instrumented knee brace were downsampled to the recording frequency of the reference system (250 Hz).

The Linear Fit Method (LFM) [21] was applied to assess the agreement between the sagittal knee angle outputs of the two measurement systems on both test sessions. The benefit of the LFM approach lies in a comprehensive analysis, which includes all data point distributions. This means the data of the whole waveforms instead of single prominent points were included into the data analysis. Using the LFM, a linear regression between the instrumented knee brace and the reference system was conducted, resulting in an amplitude scaling factor (a_1_), the vertical angular displacement (a_0_), and the linear relationship (R^2^) of the two datasets. In accordance with Iosa et al. [21], the following equations were used to compute the three parameters of the LFM with P_ref_ referring to the data of the reference system and P_Ikb_ referring to the data of the instrumented knee brace:

(1) $$a_{1}=\frac{\sum_{i=1}^{N}\left(P_{\mathrm{ref}}\left(i\right)-\bar{P_{\mathrm{ref}}})\cdot \left(P_{\mathrm{Ikb}}(i\right)-\bar{P_{\mathrm{Ikb}}}\right)}{\sum_{i=1}^{N}{\left(P_{\mathrm{ref}}\left(i\right)-\bar{P_{\mathrm{ref}}}\right)}^{2}}$$

(2) $$a_{0}=\bar{P_{\mathrm{Ikb}}}-a_{1}\cdot \bar{P_{\mathrm{ref}}}$$

(3) $$R^{2}=\frac{\sum_{i=1}^{N}\left(a_{0}+a_{1}\cdot P_{\mathrm{ref}}\left(i\right)-P_{\mathrm{Ikb}}\right)}{\sum_{i=1}^{N}{\left(P_{\mathrm{Ikb}}\left(i\right)-\bar{P_{\mathrm{Ikb}}}\right)}^{2}}$$

In an ideal scenario, the waveforms from the instrumented knee brace and reference system would be equal and the coefficients R^2^, a_1_, and a_0_ would be 1, 1, and 0°, respectively. Additionally, root mean square errors (RMSE) were calculated for each activity of the two sessions using Equation (4) with y_ref_ referring to the data of the reference system and y_Ikb_ referring to the data of the instrumented knee brace. The Wilcoxon signed rank test was used to test for differences between short- and long-term agreement with α = 0.05.

(4) $$\mathrm{RMSE}=\sqrt{\frac{\sum_{i=1}^{N}{\left(y_{\mathrm{Ikb}}-y_{\mathrm{ref}}\right)}^{2}}{N}}$$

The continuously tracked knee data was analyzed and checked for outliers beyond the limits of passive sagittal knee ROM to characterize the instrumented knee brace’s monitoring ability. Therefore, the percentages of data points outside the limits of −10° knee extension and 160° knee flexion were calculated.

## 3. Results

### 3.1. Agreement between Sensor and Reference System

**Figure 4:**
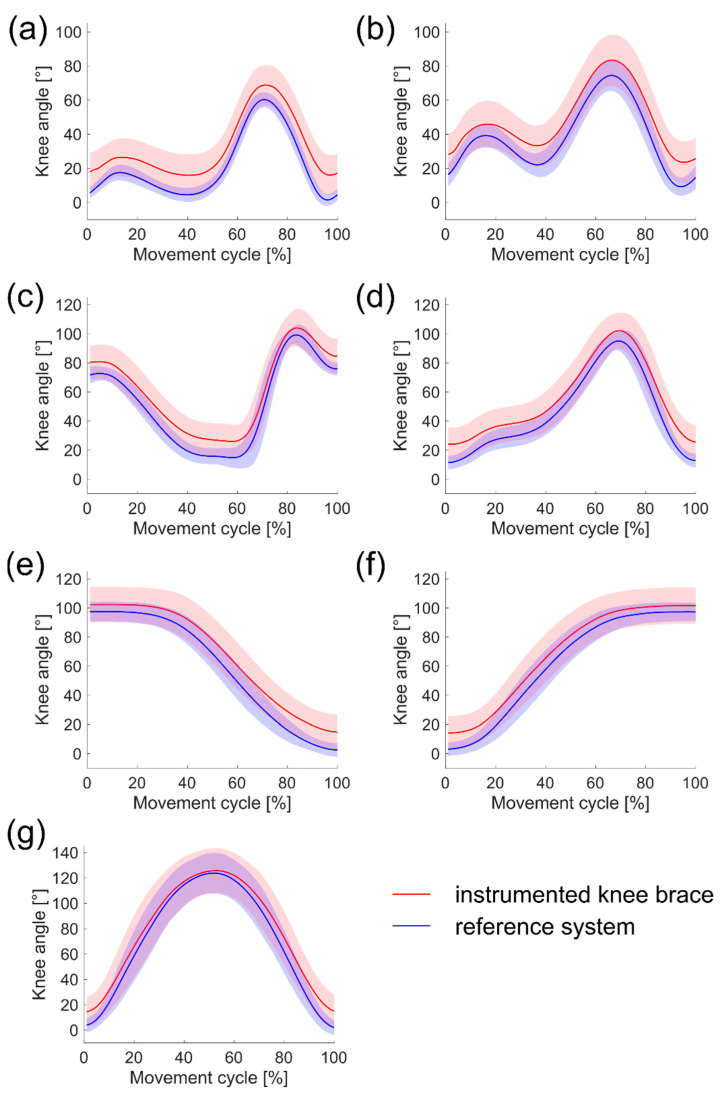
Knee angle outputs of session 1 for (**a**) walking, (**b**) jogging, (**c**) stair ascent, (**d**) stair descent, (**e**) chair rise, (**f**) sitting down, and (**g**) squatting; the red line and corresponding shaded area represent the mean knee angle and standard deviation measured by the instrumented knee brace, the blue line and corresponding shaded area represent the mean knee angle and standard deviation measured by the reference system.

The results of both test sessions are summarized in Table 1 and shown in Figure 4. There was a strong linear relationship between the measurements from the instrumented knee brace and the reference system for both sessions, with R^2^ values greater than 0.9 for all activities. The mean R^2^ values between activities ranged from 0.989 to 0.999 (*n* = 32) and 0.988 to 0.999 (*n* = 9) for S1 and S2, respectively. Amplitude scaling factors were close to the ideal output of 1 for all activities, with mean values of a_1_ ranging from 0.92 to 0.96 (*n* = 32) and 0.95 to 1.00 (*n* = 9) for S1 and S2, respectively. The mean vertical angular displacement between the instrumented knee brace and the reference system for all activities ranged from a_0_ = 7.62° to a_0_ = 10.31° (*n* = 32) in S1 and a_0_ = 0.15° to a_0_ = 3.36° (*n* = 9) in S2. This vertical offset between the two systems for S1 can also be seen for each of the ADLs examined in Figure 4. The RMSE values also reflect the results of the vertical displacement, ranging from 7.64° to 9.85° (*n* = 32) and 3.20° to 4.33° (*n* = 9) for S1 and S2, respectively.

**Figure 5:**
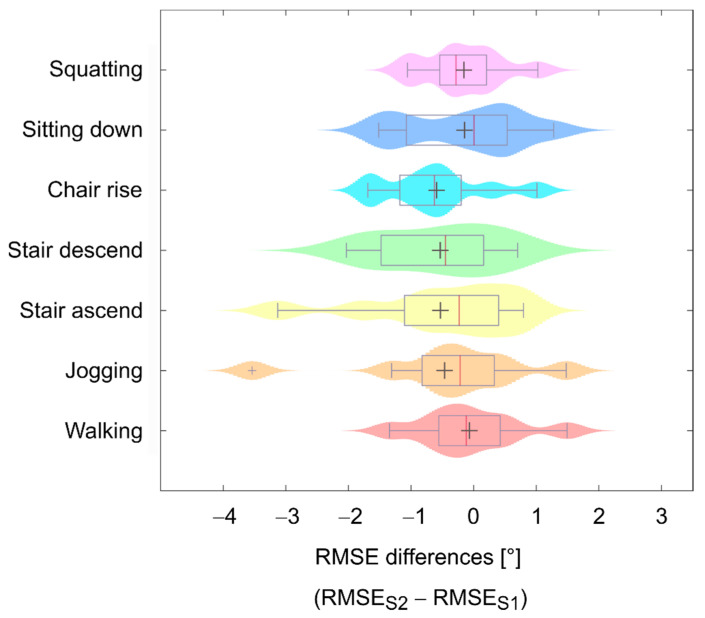
Distribution of root mean square error (RMSE) differences between session 1 (S1) and session 2 (S2) for each investigated activity obtained from the subgroup of nine participants; mean differences within the boxplots are represented by grey crosses, median differences within the boxplots are represented by red vertical lines, shaded areas represent the probability density of the data; the wider shaded areas indicate an accumulation of datapoints and narrow shaded areas, which are mostly present at the edges of each distribution, indicate scattered data points.

Similarly, in the subgroup of participants who took part in S1 and S2, no differences for R^2^ (*n* = 9, *p* = 0.820), a_1_ (*n* = 9, *p* = 0.164), a_0_ (*n* = 9, *p* = 0.910), and RMSE (*n* = 9, *p* = 0.734) were found between both sessions. This is also shown by way of example in Figure 5, where the distribution of the RMSE differences between S1 and S2 for each investigated activity is illustrated.

### 3.2. Sensor Behavior during Continuous Tracking

**Figure 6:**
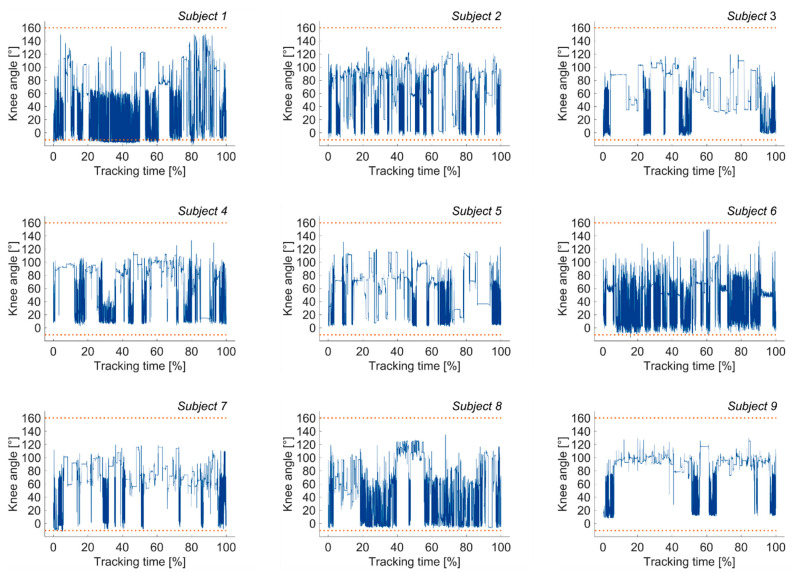
Continuously tracked sagittal knee angle data for subjects 1 through 9; the blue line represents the sagittal knee angle and the dotted orange lines mark the limits of −10° knee extension and 160° knee flexion.

The instrumented knee brace was worn for 7.1 ± 0.8 h by a subgroup of nine participants during a typical workday while their sagittal knee motion was continuously recorded. During the recording no data transmission problems occurred and complete datasets were available for outlier analysis. In three out of nine participants, outliers beyond the limits of −10° extension and 160° flexion were detected, amounting to 13.1%, 0.2%, and 0.05% of the total data for subject 1, subject 6, and subject 7, respectively (Figure 6).

### 3.3. User Acceptance of the Instrumented Knee Brace

**Figure 7:**
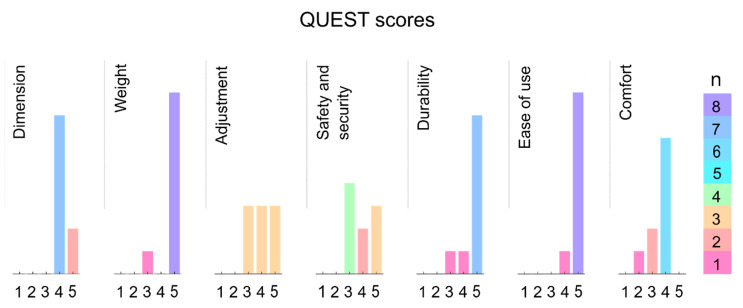
The distribution of the Quebec User Evaluation of Satisfaction with assistive Technology (QUEST) questionnaire responses as filled out by the subgroup that had worn the instrumented knee brace over the course of a day; *n* = number of responses.

The distributions of the QUEST questionnaire scores are displayed in Figure 7. The median scores for the investigated items were 4 for “Dimension,” 5 for “Weight,” 4 for “Adjustment,” 4 for “Safety and security,” 5 for “Durability,” 5 for “Ease of use,” and 4 for “Comfort” with one being least favorable and 5 being most favorable. Furthermore, participants reported a mean VAS score of 13.3 ± 5.0 for the perceived limitation in everyday life.

## 4. Discussion

This study’s objective was to validate an instrumented knee brace to measure sagittal knee motion during various ADLs. Furthermore, we aimed to assess the sensor’s potential as an instrument for continuous monitoring of sagittal knee motion, including short- and long-term applications. The study results show that the sensor’s knee angle outputs during various ADLs were in agreement with those of the reference system. Most importantly, the results were comparable between short- and long-term measurements. Furthermore, unphysiological data outputs were scarce, and sensor breakdown was not apparent. Additionally, the instrumented knee brace showed good user acceptance. However, the results also indicate some limitations of the instrumented knee brace.

As daily knee motion can be extremely variable, a sensor system needs to capture the rich variety of knee motion produced during an individual’s daily movement. The current study found a strong agreement between the instrumented knee brace and reference system as curve shape and amplitude were close to their ideal output of 1 (R^2^ > 0.9, a_1_ ≥ 0.92) for all investigated activities. The R^2^ values are similar to those obtained for IMUs [22]. The obtained RMSE values (RMSE ≤ 9.85°) are slightly higher than those reported in the literature for other wearable sensor systems that measure knee kinematics, including IMUs [23,24], flexible sensors [14,25] and potentiometers [15]. The mean angular displacement (a_0_ ≤ 10.31°) in this study shows that the instrumented knee brace slightly overestimates the reference system’s knee angle output. In particular, this overestimation seems to increase for extended knee positions (Figure 4).

There are several possible explanations for this result. The offset may be due to inaccurate palpation of anatomical landmarks or incorrect alignment of the sensor’s rotation axis during the calibration process. These methodological issues may also explain the larger standard deviations of the instrumented knee brace than those of the reference system. Another potential source of error could be the prostrate position of the participant during calibration. In addition, calibration was conducted under passive knee flexion and it is possible that knee motion behavior differed under active and weight-bearing conditions, such as walking. Further investigations regarding the role of active knee motion during calibration would therefore be worthwhile. The elastic material properties of the knee brace could also have attributed to the offset. Due to the stretch of the material, the upper and lower legs’ actual movements may not be entirely transmitted to the potentiometer. Knee motion measurement close to terminal flexion and extension is likely most vulnerable to this potential issue. Thus, absolute knee angle data must be interpreted with caution, especially towards sagittal knee angle extrema. The detected offset may be less critical for evaluating the total knee ROM or changes in total knee ROM because those measurements rely on relative rather than absolute data.

Our study showed a similar agreement between the knee brace and the reference system before and after continuous monitoring over the course of a day, as no significant differences were measured between S1 and S2 (*n* = 9, *p* ≥ 0.164). Thus, sagittal knee motion is consistently measurable with the instrumented knee brace for various ADLs even after wearing the brace for multiple hours. During the mean tracking time of 7.1 ± 0.8 h, the sensor proved to be durable, as no connectivity problems or failures in data transmission occurred. Furthermore, the recorded knee angles of nine subjects were almost entirely within the limits of physiological knee ROM, here set to −10° knee extension and 160° knee flexion, except for three subjects. The most significant aberrations were found in subject 1, where 13.1% of the total recorded data was beyond the defined limits of physiological knee ROM. Given that the instrumented knee brace and reference system showed strong agreement for subject 1 during both ADL measurement sessions in the laboratory (S1: 1.42° ≤ RMSE ≤ 2.76°, S2: 1.19° ≤ RMSE ≤ 2.64°), it is possible that the clothing worn by the participant during the continuous monitoring phase might have influenced the output data. For future studies, the assessment of participants’ passive ROM might provide individualized guidance on the limits within which knee angle data can be expected. Unfortunately, we are unable to compare our long-term results to previous findings. Although a few studies have investigated continuous sagittal knee motion during daily activities using either goniometers [17,18,26,27] or IMUs [28], there is a paucity of data on the long-term performance of those sensors.

Besides the technological and physiological challenges, we aimed to evaluate the user acceptance of the instrumented knee brace. When verbally asked, our participants did not report discomfort while wearing the instrumented knee brace during the single measurement sessions. Similarly, the median QUEST scores reached median values of 4 or 5 for all solicited questionnaire items, indicating that the participants of the subgroup were satisfied with the instrumented knee brace during continuous recording. Moreover, these participants, who wore the instrumented knee brace over the course of a day, only perceived minor limitations in everyday life (VAS score: 13.3 ± 5.0). Even though the cable connection between sensor and data logger can be a little obstructive in daily use, the brace can be easily worn under clothing and the data logger can be attached to a belt or put in a pocket. To further improve user acceptance and patient compliance for possible applications in clinical research, future iterations of the instrumented knee brace could include a wireless system solution, along with a smaller amplifier box design.

Overall, the potentiometer-based sensor system can be a suitable choice for continuous knee motion monitoring, with which it may be possible to detect changes in knee ROM throughout the day, as well as an individual’s characteristic movement patterns. Our study showed that the instrumented knee brace fulfills most requirements for knee monitoring systems, including its ability to measure sagittal knee motion during various ADLs and continuously track sagittal knee motion over several hours. Additional advantages of this knee-monitoring brace include its easy application, the sensor’s robustness to drift, and its low cost. It also does not require complex programming algorithms to obtain knee angle data.

However, a significant limitation of this sensor system is that it measures knee motion only in one plane. Inertial measurement units and flexible electrogoniometers are able to measure knee motion in multiple planes, but have disadvantages, which can include crosstalk [29,30], interference by the clothing worn over the device [31], and a recurring drift in the data after signal integration [32,33]. Notwithstanding this limitation, the instrumented knee brace can still be a potential sensor system for clinical applications. Deficits after injury are often detected in the sagittal plane and therefore sagittal knee ROM is one of the most frequently reported clinical outcome measures. However, in order to be a useful tool in clinical practice, clinical parameters measured with the sensor system will need to be identified and calculated. These should then be made available for clinicians and therapists, for example, via a mobile app, to derive meaningful insights into patient rehabilitation. Therefore, future work will need to determine which clinically significant parameters can be extracted in addition to knee ROM from the output of the instrumented knee brace.

Another potential limitation of the current potentiometer-based sensor system might be its calibration process. As mentioned above, it is a relatively involved procedure and should be simplified to minimize possible measurement errors. Optimization of the calibration process should include the investigation of other calibration poses, such as standing upright to obtain more reliable calibration results. Even so, customized calibration may continue to be necessary to accommodate the individual anthropometrics and movement behaviors of patients. As previously mentioned, clothing worn over the device could also influence output data. In addition, increased humidity, most likely occurring by sweating during physical activity, could affect the measurement accuracy of the instrumented knee brace as well. Even though we found only a few outliers in the continuously tracked data, we cannot exclude the potential impacts of clothing worn over the device and/or sweating on the signal outcome. Thus, further studies should examine possible impacts of these external factors more extensively. Furthermore, the instrumented knee brace will need to be validated in additional studies, especially if intended for use in a clinical context. Therefore, future work should include measurements on a greater number of subjects and on populations with altered knee biomechanics, such as patients with knee ligament injuries.

## 5. Conclusions

We conclude that a potentiometer embedded into a knee brace can be a suitable sensor solution for tracking knee motion in real-life environments. The sensor integration presented here proved to be durable during data tracking over several hours and was able to measure sagittal knee motion during various ADLs. A possible application of the knee brace could include monitoring knee motion after knee ligament injury to both identify patients’ individual therapeutic needs and, by employing that information, to improve rehabilitation outcomes. Though the lack of long-term sensor validation should be addressed in future work, the results of this study show promise for the broader application of wearable sensors towards understanding our movement in diverse (unsupervised) environments.

## Figures and Tables

**Table 1:** Summary of the Linear Fit Method (LFM) outputs and root mean square error (RMSE) values (mean ± SD) for session 1 (S1) and session 2 (S2); ADL = activity of daily living.

ADL	Session		a_1_	a_0_ [°]	R^2^	RMSE [°]
Walking	S1	*n* = 32	0.96 ± 0.07	8.04 ± 9.77	0.989 ± 0.005	9.09 ± 7.52
		*n* = 9	1.00 ± 0.06	0.33 ± 2.75	0.988 ± 0.005	3.38 ± 1.14
	S2	*n* = 9	1.00 ± 0.06	0.15 ± 3.48	0.988 ± 0.005	3.31 ± 1.34
Jogging	S1	*n* = 32	0.95 ± 0.05	8.73 ± 9.45	0.989 ± 0.006	8.81 ± 6.92
		*n* = 9	0.98 ± 0.04	1.33 ± 2.82	0.989 ± 0.004	3.87 ± 1.26
	S2	*n* = 9	0.97 ± 0.04	1.45 ± 3.13	0.991 ± 0.003	3.40 ± 1.27
Stair ascent	S1	*n* = 32	0.94 ± 0.04	9.51 ± 9.27	0.998 ± 0.002	8.39 ± 6.82
		*n* = 9	0.96 ± 0.03	2.45 ± 2.94	0.997 ± 0.003	3.73 ± 1.48
	S2	*n* = 9	0.96 ± 0.03	1.93 ± 2.79	0.998 ± 0.001	3.20 ± 1.64
Stair descent	S1	*n* = 32	0.95 ± 0.03	8.15 ± 8.99	0.995± 0.003	8.45 ± 6.69
		*n* = 9	0.97 ± 0.02	1.23 ± 3.03	0.994 ± 0.003	3.84 ± 1.45
	S2	*n* = 9	0.97 ± 0.03	0.89 ± 2.90	0.994 ± 0.003	3.31 ± 1.52
Chair rise	S1	*n* = 32	0.92 ± 0.05	10.31± 9.42	0.999 ± 0.002	8.69 ± 6.40
		*n* = 9	0.95 ± 0.06	3.63 ± 3.35	0.998 ± 0.002	4.78 ± 2.02
	S2	*n* = 9	0.95 ± 0.05	3.36 ± 2.95	0.998 ± 0.002	4.19 ± 1.82
Sitting down	S1	*n* = 32	0.94 ± 0.05	7.62 ± 9.27	0.999 ± 0.001	7.64 ± 5.64
		*n* = 9	0.96 ± 0.05	1.00 ± 3.44	0.999 ± 0.001	4.20 ± 1.14
	S2	*n* = 9	0.96 ± 0.05	0.72 ± 3.01	0.999 ± 0.001	4.05 ± 2.32
Squatting	S1	*n* = 32	0.94± 0.05	8.92 ± 11.51	0.998 ± 0.002	9.85 ± 6.93
		*n* = 9	0.95 ± 0.04	2.31 ± 2.50	0.998 ± 0.001	4.49 ± 2.33
	S2	*n* = 9	0.95 ± 0.04	1.83 ± 2.94	0.998 ± 0.001	4.33 ± 2.37

## Data Availability

The data presented in this study are available on request from the corresponding author.
